# The Effect of Calorie Restriction on Protein Quality Control in Yeast

**DOI:** 10.3390/biom13050841

**Published:** 2023-05-15

**Authors:** Petter Uvdal, Sviatlana Shashkova

**Affiliations:** Department of Physics, University of Gothenburg, 405 30 Göteborg, Sweden; petter.uvdal@physics.gu.se

**Keywords:** yeast, *Saccharomyces cerevisiae*, protein quality control, carbon metabolism, calorie restriction, degradation, autophagy, Hsp104, misfolded proteins, protein aggregation, neurodegenerative diseases, age-related diseases, stress response

## Abstract

Initially, protein aggregates were regarded as a sign of a pathological state of the cell. Later, it was found that these assemblies are formed in response to stress, and that some of them serve as signalling mechanisms. This review has a particular focus on how intracellular protein aggregates are related to altered metabolism caused by different glucose concentrations in the extracellular environment. We summarise the current knowledge of the role of energy homeostasis signalling pathways in the consequent effect on intracellular protein aggregate accumulation and removal. This covers regulation at different levels, including elevated protein degradation and proteasome activity mediated by the Hxk2 protein, the enhanced ubiquitination of aberrant proteins through Torc1/Sch9 and Msn2/Whi2, and the activation of autophagy mediated through *ATG* genes. Finally, certain proteins form reversible biomolecular aggregates in response to stress and reduced glucose levels, which are used as a signalling mechanism in the cell, controlling major primary energy pathways related to glucose sensing.

## 1. Overview

Yeast was the first organism in which the genes responsible for increased lifespan were identified [1]. Since then, yeast has become an invaluable model system to study pathological conditions and ageing, including through the expression of human proteins involved in neurodegenerative disorders, such as Alzheimer’s, Parkinson’s and Huntington’s diseases [2,3,4]. The budding yeast *Saccharomyces cerevisiae* has been widely used in studies of how different nutritional environments affect proteostasis and the relationship between glucose metabolism and the accumulation of misfolded proteins [5,6,7,8,9,10,11,12,13,14,15,16,17,18,19]. In this review, we summarise the current knowledge on the connection between protein aggregation and the depletion of glucose and how it is controlled through glucose signalling pathways. We first discuss how protein aggregates are affected by calorie restriction in pathological conditions. Then, we examine how recognising and subjecting aberrant proteins to the Protein Quality Control (PQC) system, protein refolding and turnover are controlled by stress resilience genes, e.g., the activation of stress response elements (STREs) in the genome. Finally, we look into how protein aggregates are used in the cell as a signalling mechanism to control primary energy pathways.

## 2. Yeast as a Model System for Ageing, Neurodegenerative Diseases and Stress

In yeast, aberrant proteins form protein aggregates, which are prevalent in dysfunctional and pathological conditions that arise due to ageing, diseases or certain mutations, such as the expression of human disease-associated proteins, etc. [20,21]. Protein aggregates are also accumulated under specific environmental stress conditions, e.g., in response to heat shock or due to ethanol, oxidative or osmotic stress [21,22,23]. Certain insoluble amyloidogenic protein aggregates are associated with neurodegenerative diseases [4]. For example, Huntington’s disease (HD) causes the aggregation of huntingtin (Htt) fragments containing repeating units of polyglutamine (PolyQ) at the N-terminus [3]. Amyloid-β and tau proteins are involved in the progression of Alzheimer’s disease (AD), while α-, β- and γ-synucleins are associated with Parkinson’s disease (PD) [2,3,4,24]. Similarly, during ageing, a consequent increase in intracellular H_2_O_2_ leads to damage in native proteins [25,26]. In most cases of soluble and insoluble aggregates, disaggregation is dependent on Hsp42, a small heat shock protein that recruits the chaperones Hsp104/Hsp70 [22,26]. The Hsp104 disaggregase is widely used as a reporter for misfolded proteins, as it binds to stress-induced protein aggregates and mediates their refolding or degradation [26,27,28,29,30]. Accumulating protein aggregates are shielded in inclusions, and Hsp104 sequesters the insoluble aggregates into insoluble amyloid protein deposits (IPODs), while soluble aggregates are transported to the juxtanuclear quality control (JUNQ) compartment or intranuclear quality control (INQ) site [31]. In the JUNQ and INQ compartments, proteasomes are prevalent, which ensures the degradation of soluble constituents of protein aggregates, i.e., aberrant and misfolded proteins [32]. Inclusions in both protein deposits increase with the progression of the state of neurological disease or with age [23]. During cell division, damaged proteins are typically spatially separated to remain in the mother cell, keeping the newly produced daughter cell free of damage [33]. This spatial quality control is mediated through asymmetry-generating genes (AGGs), such as *vac17* [33]. Disruptions in the interaction between Hsp104 and endocytic vesicle trafficking, e.g., through *vac17*∆, impedes such asymmetry. This leads to the inheritance of damaged proteins yet also increases the replicative lifespan of the mother cell, as misfolded protein aggregates are consistently removed and transferred to daughter cells [33].

## 3. The Effect of Calorie Restriction on Protein Aggregation in Yeast

A low glucose environment has been linked to the accumulation of misfolded proteins in yeast, as it invokes a stress response [15,16]. A low glucose concentration in the yeast medium has been accepted as a calorie restriction (CR) condition [34]. Upon CR, yeast adapts to the new conditions by decreasing the biosynthesis of macromolecules, which, in the long term, extends the replicative and chronological lifespan (RLS and CLS, respectively) [5,12,13]. CR has been shown to temporarily increase the production of reactive oxygen species (ROS), which leads to the activation of hormesis [1,35,36]. Moreover, CR inhibits target of rapamycin complex 1 (Torc1) of the target of rapamycin (TOR) pathway. This and the consequent deficiency of the Mtl1 protein trigger the formation of stress granules [37,38]. The inhibition of Torc1 also leads to the activation of the stress response and autophagy [39]. Finally, glucose deprivation decreases the rate of protein glycosylation and disrupts Ca^2+^ homeostasis, which results in an increase in the number of unfolded proteins in the endoplasmic reticulum (ER) and the activation of the unfolded protein response (UPR) [36,40,41,42].

Using single-cell fluorescent microscopy, it has been shown that glucose starvation elevates the number of yeast cells with Hsp104-bound aggregates within 90 min of low glucose exposure [16]. This is an indication that such conditions moderately increase the protein aggregation rate. This is exemplified by the fact that the number of cells with Hsp104-bound aggregates was lower than in other stress conditions, such as heat shock or osmotic stress. Nonetheless, a two-hour pre-adaptation to LiCl or NaCl resulted in fewer cells with aggregates upon glucose starvation. It was also shown that glucose limitation mitigates the negative effects of LiCl on cell survival, suggesting that adaptation to low glucose conditions is related to other stress pre-adaptations [16]. This is consistent with the fact that CR induces mild stress in the cell, as the levels of ROS are increased through the inactivation of catalase activity [35]. Yet, cells adapt to mild stress through the activation of hormesis, which consequently improves resistance to other stress factors.

Similarly, a high level of glucose also induces a mild stress response in the cell due to the proteotoxic product of glycolysis, the compound methylglyoxal (MG) [15,43]. MG increases ROS, which interferes with the PQC system and leads to an increase in Hsp104-bound protein aggregates and inclusions. In non-stress conditions, MG induces a metabolic stress response in the cell, which activates a mild hormetic response [15].

During glucose starvation, Hsp104-dependent clearance of protein aggregates is impaired through the depletion of ATP [17]. It has been shown that protein aggregates disappear within minutes of reintroducing *S. cerevisiae* from a low to high glucose-concentration environment as ATP levels are restored to normal. Impaired mitochondrial PQC through the deletion of the ATP-dependent Lon protease homologue, Pim1, has a similar effect and results in the aggregation of oxidised proteins and decreased proteasome activity [18,19]. Therefore, normal energy homeostasis is necessary for the clearance of stress-induced aggregates, yet, over time, yeast can adapt to the new environment.

## 4. Adaptation to Calorie Restriction and the Consequent Effect on Protein Turnover

Several hypotheses have been proposed on the reasons for the extension of replicative lifespan potential. These include increased protein turnover, fewer protein aggregates due to the overexpression of chaperones or fewer misfolded proteins as the result of decreased ribosome biogenesis and translation, and a general decrease in protein synthesis [5,14,44,45]. The next topic to be addressed is how CR affects the degradation rate of aberrant proteins. The primary players responsible for the degradation of aberrant intracellular proteins are the Ubiquitin Proteasome System (UPS), autophagy and ER-associated degradation (ERAD) [7,13,46].

### 4.1. The Ubiquitin Proteasome System

The UPS regulates intracellular levels of aberrant proteins by degrading them; hence, the UPS capacity largely impacts damage accumulation [13,14]. The ubiquitination of abnormal proteins is essential for their subsequent recognition and degradation by the proteasome [47].

Overall, protein turnover has been suggested to be higher under CR conditions [5,6]. It has been shown that the number of polyubiquitinated proteins is significantly greater in *S. cerevisiae* cells grown with calorie excess (CE) than in those cultivated with CR. Interestingly, in aged yeast, it seems to be the opposite [13]. Although the proteolytic activity of the UPS proteasome becomes elevated during ageing, proteasome-mediated degradation suffers a progressive loss of function. This occurs despite the enhanced expression of genes necessary for an increase in UPS capacity, e.g., genes controlling proteasome subunit biogenesis. It has been suggested that this is a result of elevated protein oxidation, which impairs ubiquitin enzymes that are essential for proteasome-mediated degradation [48]. Similarly, it has been proposed that, in older cells, the ubiquitin-activating E1 enzyme is impaired by the increased oxidative intracellular environment [13].

The oxidative environment, i.e., the accumulation of intracellular H_2_O_2_, also damages native proteins and leads to protein aggregation [21,26]. Since CR counteracts the accumulation of oxidative species during ageing, it has been suggested that CR increases the number of ubiquitinated proteins in aged cells, which is related to a reduction in protein aggregates [13].

The ubiquitin-protein ligase Ubr2 regulates the turnover of the proteasome transcription factor Rpn4, which is essential for the fine-tuning of proteasome activity. The deletion of the *UBR2* gene leads to the increased capacity of UPS, which results in an increase in protein turnover and extends lifespan [14]. Furthermore, an elevated UPS capacity enhances the clearance of aggregates of toxic huntingtin fragments, Htt103Q, while the non-toxic (Htt25Q) protein assemblies remain unaffected. Hence, the enhanced UPS capacity leads to elevated proteasome activity and lifespan extension, which is distinct from lifespan extension through dietary restriction and the inhibition of Tor1 [14].

### 4.2. The Role of Autophagy and ERAD

**Figure 1:**
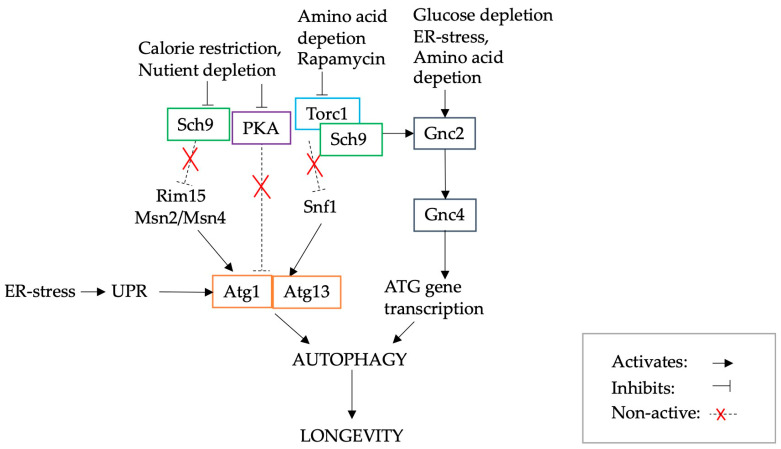
A schematic diagram illustrating the pathways implicated in activation of autophagy, re-illustrated from Tyler et al. [7].

Autophagy is a catabolic degradation process whereby damaged intracellular proteins and defective organelles are transported in membrane vesicles and degraded in the lysosome/vacuole [7,39,49,50,51,52]. Autophagy is activated through stress, or directly by the UPR, and, in yeast, is known to be responsible for the increase in both CLS and RLS [53]. The main conditions responsible for the activation of autophagy are calorie restriction, nutrient depletion, rapamycin, amino acid depletion, glucose depletion, ER stress or altered tRNA homeostasis [7,54]. Calorie, nutrient and amino acid depletion lead to the inhibition of nutrient signalling pathways, including protein kinase A (PKA) and TOR/Sch9, which activate autophagy-related genes (*ATG*) and therefore lead to increased autophagy [39,50,51]. It has also been suggested that ER stress activates *ATG* genes, e.g., those encoding Atg1 and Atg13 proteins, through sucrose non-fermenting protein kinase (Snf1), the yeast homologue of mammalian AMPK [55]. Glucose and amino acid depletion also directly increase autophagy through the activation of the Gnc2 protein and consequently Gnc4, which leads to the transcription of *ATG* genes. Furthermore, autophagy is especially important in pathological conditions, such as neurodegenerative and age-related diseases, where, e.g., rapamycin and latrepirdine have been shown to enhance autophagy and hence reduce amyloid-β aggregates in models of Alzheimer’s disease [54]. A schematic diagram of the activation of autophagy and relevant pathways is presented in Figure 1.

In terms of ER stress, CR leads to an increase in unfolded proteins in the ER, which in turn leads to the activation of the UPR and consequently autophagy in an Atg1-dependent manner [36,40,41,42,56]. A disruption of the UPR also increases the number of misfolded proteins in the ER in response to proteotoxic stress [56,57]. The chromatin remodelling complex SWI/SNF has been suggested to be necessary for ER stress signalling upon heat and proteotoxic stress [57]. For example, deletions within this complex have been shown to increase misfolded protein accumulation in the ER in response to cadmium [57,58]. Misfolded proteins can also be degraded through ER-associated degradation (ERAD), whose functionality is necessary for a normal lifespan [56]. ERAD is activated by ER stress and is possibly used when autophagy is impaired. Yet, little is known about the physiological relevance of ERAD [46,59].

Previously, it has been shown that the inhibition of TOR1, a subunit of the TORC1 kinase, activates autophagy in yeast [47,48,49,50,51]. In *S. cerevisiae*, TORC1 works in parallel with the UPR, where TORC1 inactivation mediates sensitivity to ER stress [60]. The abnormal activation of TORC1 has been shown to lead to higher MG levels and an increased number of protein aggregates, as well as lower proteasome activity. Such overactivation is induced by the chaperone Hsp31 and mediated through Sfp1, a transcription factor involved in ribosomal biogenesis [43]. Moreover, the deletion of TOR1 increases cellular fitness and extends lifespan in yeast through enhanced autophagy [7,14].

## 5. Mediated Adaptation to Glucose Starvation through Glucose Signalling Pathways and the Corresponding Effect on PQC

Genetic modifications in yeast have been a promising approach to evaluating the role of specific pathways in the stress response [5,6,8,9,10,11]. In low glucose conditions, yeast cells switch from fermentation to respiration (Crabtree effect) and hence redirect glucose utilisation [5]. This slows down metabolic processes and decreases the biosynthetic burden yet elevates proteasome activity and activates autophagy. In yeast, the effects of various pathways, such as cAMP-PKA and TOR, through different proteins, including Hxk2, Gpa2/Gpr1, Sch9, Snf1 and Msn2, on PQC have been studied [5,6,8,10,11,61]. For example, yeasts with impaired glucose sensing through the deletion of Gpa2 and Gpr1, which are involved in the cAMP-PKA pathway, exhibit an extended lifespan regardless of the glucose concentration in the medium [5].

**Figure 2:**
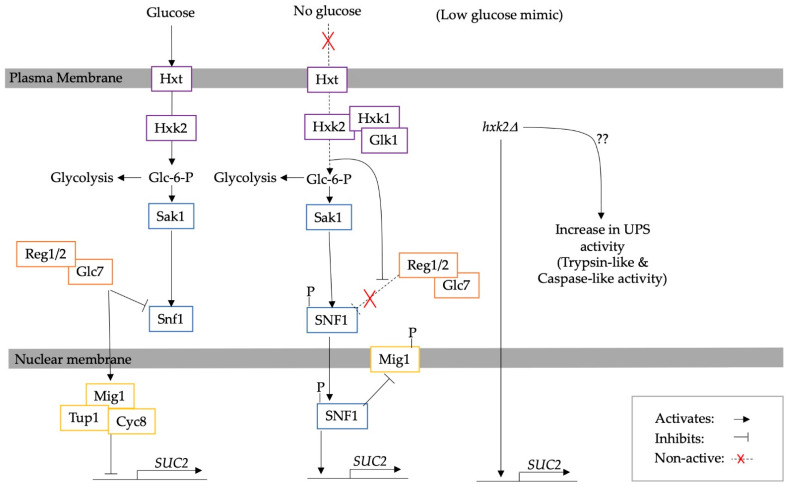
A diagram illustrating the upregulation of UPS activity due to *hxk2*∆. Hxk2 signals the inhibition of *SUC2* in response to glucose. Re-illustrated from Bendrioua et al. [64]. However, *hxk2*∆ increases the amount of ATP regardless of glucose availability and respiration/fermentation in yeast. It also upregulates protein turnover by increasing proteasome activity [6].

Similarly, hexokinase 2 (Hxk2) is involved in central carbon metabolism and facilitates the repression of genes essential for the utilisation of non-glucose carbon sources, such as *SUC2*, via the transcriptional repressor Mig1, one of the targets of Snf1 [62,63]. *hxk2*∆ works as a calorie restriction mimic and robustly increases RLS [6,61]. However, reduced proteasome activity abrogates this effect. Hence, an interconnected link between proteasomes, Hxk2 and the Snf1 pathway has been proposed [61]. The *hxk2*∆ mutation also results in an increase in the ATP content, regardless of respiration or fermentation, while at the same time enhancing proteasome activity (increased chymotrypsin-like and caspase-like activity). A schematic diagram of the intervention is presented in Figure 2. However, in wild-type cells, low glucose conditions do not seem to increase proteasome activity. This could be contradictory to previously discussed studies; however, increased proteasome activity has only been shown for aged cells in CR [6].

**Figure 3:**
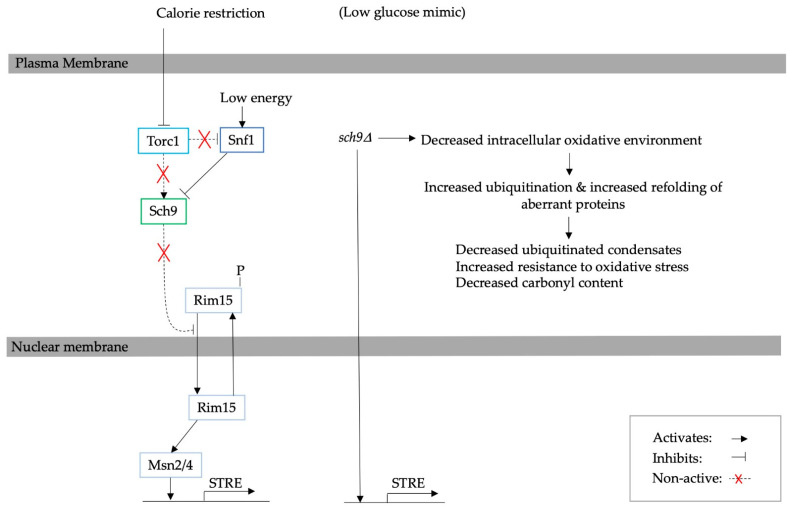
A diagram explaining the effect of *sch9*∆ [10]. Sch9 depletion activates Rim15 and Msn2/4, which in turn activate STRE [65,66]. The STRE decreases the build-up of H_2_O_2,_ which reduces the oxidisation of intracellular proteins, including newly synthesised proteins and their subsequent ubiquitination. Hence, there is an improved capacity to ubiquitinate and refold misfolded proteins.

In yeast, the TOR, PKA and Sch9 kinases are regulated by nutrient availability. *tor1*∆ and *sch9*∆ increase lifespan; however, Gcn4 is also needed for lifespan extension through the activation of *ATG* genes [14]. The inhibition of Sch9 has been shown to be a result of TORC1 deactivation and mimics nutritional depletion and calorie restriction. The deletion of *SCH9* has been shown to reduce the number of ubiquitinated proteins and carbonyl content in the log growth phase of the yeast *S. cerevisiae*, without affecting UPS activity or autophagy [10]. However, no shortage of free ubiquitin availability was observed that could have caused a decrease in ubiquitination. At the same time, the *SCH9* deletion cells showed more Hsp104 aggregates compared to the wild-type strain. More specifically, Sch9 depletion activates stress response regulators, such as STREs, decreases the accumulation of H_2_O_2_ and consequently reduces the oxidisation of intracellular proteins. An increase in the oxidative environment impairs the ubiquitination of intracellular proteins, including newly synthesised proteins. The oxidation of newly synthesised proteins could also cause misfolding [21,26]. Furthermore, a reduction in the oxidative environment seems to improve the capacity to refold misfolded proteins [10]. This finding suggests that the deletion of *SCH9* improves the refolding of aberrant proteins, which is illustrated in Figure 3.

**Figure 4:**
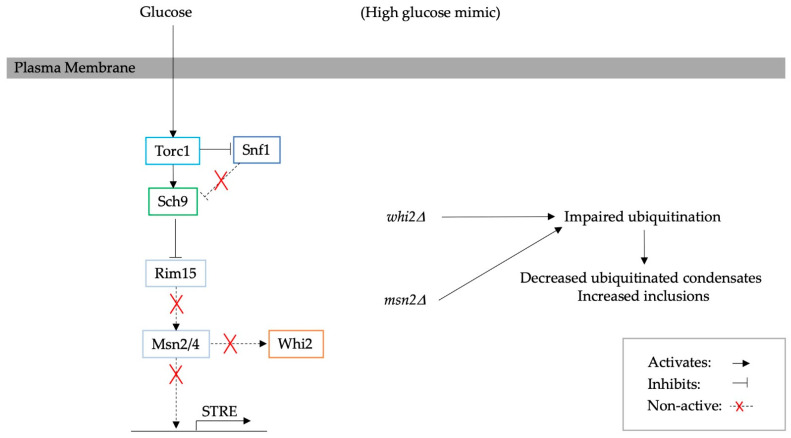
A diagram illustrating the effect of *msn2*∆/*whi2*∆ [8]. Msn2 activates Whi2 and STRE [65,66]. Through the depletion of Msn2 and consequently Whi2, the ubiquitination process is inhibited [8]. Previously, it has been suggested that the stress response ameliorates the ubiquitination process by decreasing the levels of reactive oxidative species [10].

The overexpression and deletion of *MSN2* have also been shown to directly influence proteostasis. In high glucose conditions, Msn2 is inhibited by both the Torc1/Sch9 and cAMP-PKA pathways [67]. The deletion of *MSN2* hinders the Msn2-mediated stress response, which mimics high glucose conditions. Interestingly, this mutation also leads to an increase in the number of inclusions formed by protein aggregates yet causes a decrease in levels of ubiquitinated proteins. For instance, in *msn2*∆ cells, Guk1-7-GFP, a temperature-sensitive construct that is degraded when cells are shifted to 37 °C, becomes less ubiquitinated compared to the wild type [8]. Therefore, *msn2*∆ increases the stability of heat-induced protein aggregates, such as Guk1-7-GFP. Interestingly, Qie B. et al. suggested that the deletion of Sch9, the upstream regulator of Msn2, enhances the removal of ROS, which leads to fewer ubiquitinated proteins [10]. Msn2 also regulates the *WHI2* gene, and *whi2*∆ has been shown to have the same effect on ubiquitination as *msn2*∆. Thus, *MSN2/WHI2* are involved in proteostasis, as they are connected to the improved ubiquitination of aberrant proteins and are essential for the cell’s ability to refold and/or degrade misfolded proteins [8]. A schematic diagram of the pathway controlling protein homeostasis through Msn2 and Whi2 is illustrated in Figure 4.

However, the Msn2-mediated stress response impairs resistance to toxic amino acid analogues [9]. Under normal conditions, the overexpression of this protein results in higher levels of ubiquitin-conjugated proteins, suggesting the enhancement of the ubiquitination process. On the other hand, the overexpression of Msn2 has been shown to lead to an increase in Gnp1 protein expression. Gnp1, together with deubiquitinating enzymes (DUBs), deplete free ubiquitin levels in the presence of azetidine-2-carboxylic acid (AZC), as well as other toxic amino acid analogues [9].

**Figure 5:**
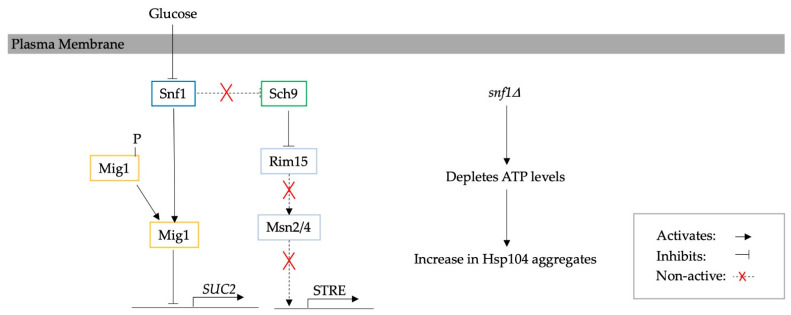
A diagram illustrating the effect of Snf1 [64,65,66]. Specifically, *snf1*∆ depletes ATP levels, which increases the number of Hsp104-bound aggregates while not affecting the number of stress granules [11].

The central carbon metabolism pathway that has recently been implicated in the effect on protein aggregates is SNF1 [11]. A diagram showing the effect of Snf1 is illustrated in Figure 5. Specifically, *snf1*∆ has been found to impair ATP homeostasis, in synergy with the deletion of adenylate kinase, Adk1, the key enzyme that synthesises ATP and AMP, and the de novo purine-synthesising transcription factor Bas1 [68]. However, the deletion of the transcription factor Mig1 does not seem to affect ATP levels, and it was hypothesised that Snf1 controls ATP levels through other targets. Deleting one or more of the genes that regulate ATP content within the cell results in an increase in the number of Hsp104-bound aggregates. However, the assembly of the stress granule marker, Pab1, was not observed, indicating that protein aggregation and stress granule formation are regulated by different mechanisms [11].

Communication between proteostasis and metabolic networks remains to be elucidated. In yeast, Snf1 is regulated by glucose availability and through Torc1. It has previously been found that Snf1 may be responsible for the formation of protein aggregates by modulating ATP content, while the overexpression of chaperones, such as Hsp104, would decrease the accumulation of protein aggregates through disaggregation and refolding [11,22].

**Figure 6:**
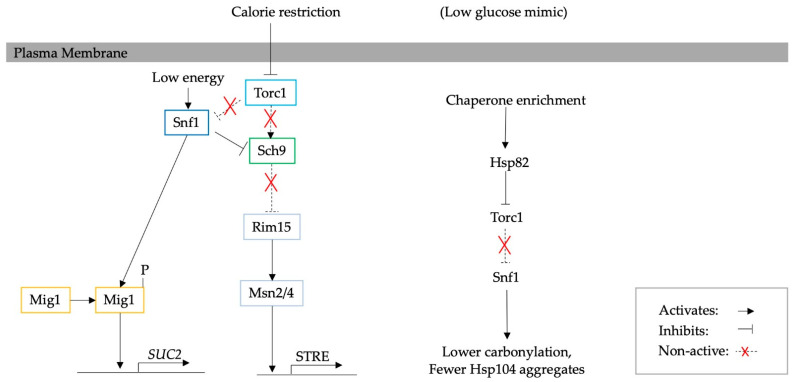
A diagram illustrating a calorie restriction phenotype induced by chaperone enrichment and inhibition of Torc1. The calorie restriction phenotype resulted in a decrease in Hsp104 aggregates and carbonyl content through the activation of Snf1 [44]. It has been shown that *snf1*∆ impairs the stability of ATP levels and thus increases the formation rate of Hsp104-bound aggregates [11]. This supports the previously reported effect of the calorie restriction phenotype on protein aggregation [10].

Another factor affecting proteostasis is chaperone enrichment, which induces the starvation phenotype through the deactivation of Torc1 [44]. An illustrative diagram of the process can be seen in Figure 6. Chaperone enrichment strains (ChESs) exhibit lower levels of protein carbonylation and fewer Hsp104-bound protein aggregates. Moreover, the activation of Snf1, which is characteristic of the starvation phenotype, was observed upon chaperone enrichment. This seems to be due to the negative regulation of Snf1 by Torc1, which senses chaperone enrichment through the Hsp82 protein. This leads to altered metabolic features and mitochondrial activity and an increase in the RLS [44]. This was interpreted as a paradigm shift in the role of proteostasis and ageing, where the modulation of misfolded proteins could also be sensed by Torc1 and impact metabolic pathways.

## 6. Reversible Aggregates as a Signalling Mechanism

In response to glucose starvation, 33 proteins have been observed to form reversible cytoplasmic foci in yeast cells. These proteins form insoluble clusters that transition to soluble upon the readdition of nutrients [69]. Some of these protein aggregates also function as signalling mechanisms controlling major energy pathways [70]. For example, Snf1 (AMPK) has been suggested to be regulated by reversible punctate foci of Std1 [71]. This process is partially controlled by glucose through the protein kinase Vhs1, a novel component acting upstream of Snf1 [71,72]. Starvation conditions have been shown to facilitate TORC1 disassembly, with the main complex component, Kog1, translocating into a single body near the vacuole in an Snf1-dependent manner. This build-up of Kog1 has been proposed to serve as a mechanism opposing the immediate reactivation of TORC1 when glucose becomes available [73].

**Figure 7:**
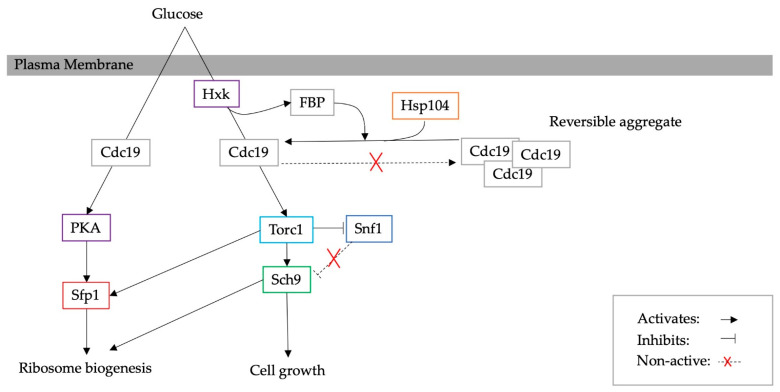
Reversible aggregation of Cdc19 regulates stress-related pathways [70,75].

Similarly, the pyruvate kinase Cdc19 forms functional reversible amyloid aggregates [70,74]. The solubility of Cdc19 has been shown to regulate both Torc1 and Ras/PKA, which control Sch9 and Sfp1, respectively, and consequently cell growth and ribosome biogenesis [74]. Mutant cells with irreversible Cdc19 aggregates seem not to be able to restore their growth after heat shock. Cdc19 aggregation is promoted by the glycolytic metabolite fructose-1,6-bisphosphate (FBP); however, recently, this interaction has been suggested to play a mechanistic role in the re-solubilisation of aggregates by recruiting Hsp104 [75]. A schematic diagram is illustrated in Figure 7.

## 7. Summary and Future Outlook

**Figure 8:**
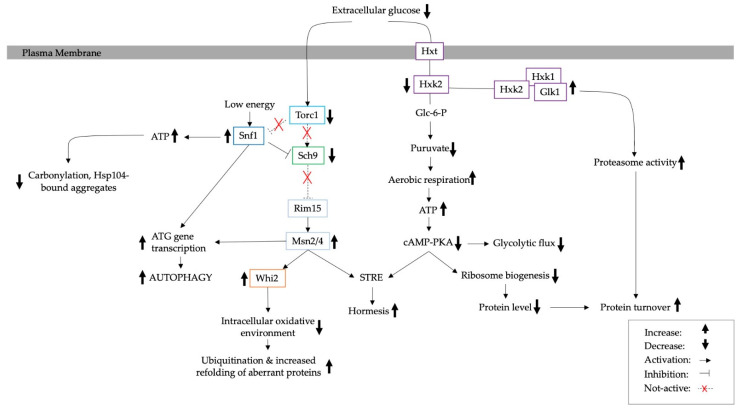
A diagram illustrating internal changes due to adaptation to calorie restriction [5,6,7,8].

In summary, protein aggregates accumulate in pathological states and in response to stress [2,21,22,23,70]. CR has been shown to contribute to the slower clearance of protein aggregates due to the depletion of ATP [17]. However, over time, cells adapt to these conditions through (a) the activation of STRE, which in turn increases proteasome activity, potentially mediated through *HXK2*, (b) the enhanced ubiquitination of aberrant proteins by Torc1/Sch9 and Msn2/Whi2, (c) the Snf1-dependent regulation of ATP levels and (d) the activation of autophagy via *ATG* genes (Figure 8) [6,7,8,9,10,11,61]. However, the regulatory mechanisms of the primary energy pathways continue to be overturned, as it has recently been found to be controlled by chaperone overexpression and reversible aggregates [44,70].

However, due to the complex relation between primary protein degradation pathways and energy utilisation, as well as a high level of crosstalk between the glucose signalling networks involved [76], more research is needed on PQC and aggregate accumulation as a response to limited glucose availability. Additional future research topics also include the transmission of stress adaptation to daughter cells. For example, stress-induced epigenomic alterations, e.g., features of Msn2, have been shown to be transferred to offspring cells [77]. The similarity of analogous genes in other organisms, their effects and functions, as well as whether adaptation to stress through regulatory mechanisms is conserved in other organisms, remain to be elucidated. For instance, pathways controlled by AMPK homologues participate in the lifespan extension of different organisms, including mammals, nematodes and yeast [78,79,80]. However, in a specific case of mutant rhodopsin, AMPK activation has been shown to accelerate photoreceptor degenerative disease in animal models [81]. It has been shown that the TOR pathway is essential for proper growth and cell division in various models, ranging from yeast to mice; however, it is also involved in the development of numerous diseases, such as diabetes, neurodegenerative disorders, etc. [82,83].

Moreover, glucose metabolism in the brain has been proposed to play a crucial role in the development of Alzheimer’s, Parkinson’s and Huntington’s diseases [84,85,86]. As protein aggregation is one of the hallmarks of these disorders, revealing the effects of calorie restriction and overall altered glucose metabolism on proteostasis and PQC in particular is a steppingstone towards better understanding the disease origin and progression. Providing such insights will also suggest novel ideas and strategies for therapeutic treatments. Protein aggregation and metabolism have also been shown to play an important role in other diseases, including cancer, diabetes, atherosclerosis, etc. [87,88,89,90,91,92]. Therefore, the topic of energy-regulatory pathways remains relevant for future fundamental scientific and medical research, providing more insights into the mechanisms relating PQC to calorie-restriction-induced metabolic stress in the context of longevity and ageing.

## Data Availability

No new data were created or analysed in this study. Data sharing is not applicable to this article.
